# Vertebral Sarcoidosis Mimicking Metastatic Disease in a Patient With Prior Melanoma: A Diagnostic Challenge

**DOI:** 10.7759/cureus.115404

**Published:** 2026-08-29

**Authors:** Andy A Garcia Salazar, Carmen Bárbara Alfonso García, Juan L Filiberto Arevalo, Idalberto Luis Fernandez Eng, Arianna Yakubu

**Affiliations:** 1 Internal Medicine, Universidad de Ciencias Medicas de Camaguey, Camaguey, CUB; 2 General Practice, Centro de Salud Benalua, Alicante, ESP; 3 Emergency Medicine, Universidad De Ciencias Medicas de Camaguey, Camaguey, CUB; 4 Emergency Medicine, Hospital Universitario de la Ribera, Valencia, ESP; 5 Hematology, Universidad de Ciencias Medicas de Camaguey, Camaguey, CUB

**Keywords:** imaging-pathology discordance, multifocal vertebral lesions, osseous sarcoidosis, sarcoidosis, vertebral sarcoidosis

## Abstract

Sarcoidosis is a multisystemic granulomatous disease that most commonly affects the lungs and intrathoracic lymph nodes. Osseous involvement is uncommon, and vertebral disease is particularly rare, often mimicking metastatic malignancy and posing a significant diagnostic challenge.

We report the case of a 51-year-old woman with a history of resected melanoma who was incidentally diagnosed with sarcoidosis following abnormal findings on screening mammography. Subsequent evaluation revealed pulmonary involvement. Months later, she developed acute back pain following minor trauma, and magnetic resonance imaging (MRI) demonstrated multifocal vertebral marrow lesions concerning for metastatic disease. However, computed tomography (CT) failed to demonstrate corresponding structural abnormalities.

Definitive diagnosis was established through vertebral biopsy, which revealed noncaseating granulomatous inflammation consistent with sarcoidosis, with no evidence of malignancy or infection. The patient demonstrated clinical improvement with corticosteroid and immunosuppressive therapy.

This case highlights the importance of considering sarcoidosis in the differential diagnosis of vertebral lesions, particularly in patients with a history of malignancy, and underscores the need for histopathological confirmation in cases with discordant imaging findings.

## Introduction

Sarcoidosis is a systemic granulomatous disorder of unclear etiology characterized histologically by the formation of noncaseating granulomas. Although virtually any organ system may be affected, pulmonary and intrathoracic lymph node involvement represent the most common manifestations of the disease [1,2]. Extrapulmonary involvement is also well-recognized and may affect the skin, eyes, heart, nervous system, and musculoskeletal system, contributing to the considerable heterogeneity of its clinical presentation [1,2].

Osseous sarcoidosis is relatively uncommon, reported in approximately 3-13% of patients. Although axial skeletal involvement was historically considered uncommon, more recent imaging-based studies have identified the spine as a frequent site of involvement among patients with osseous sarcoidosis [3]. Vertebral sarcoidosis presents an important diagnostic challenge because its radiologic appearance may resemble malignant, infectious, or other inflammatory processes [3-5].

This diagnostic overlap becomes particularly relevant in patients with a previous history of malignancy, in whom new multifocal vertebral lesions may understandably raise concern for metastatic recurrence. Discordance between imaging modalities may further complicate the diagnostic process and emphasize the importance of integrating clinical history, imaging findings, and histopathologic evaluation before establishing a definitive diagnosis.

We present a case of vertebral sarcoidosis in a 51-year-old woman with a history of resected melanoma who developed multifocal vertebral marrow lesions initially concerning for metastatic disease. The discrepancy between magnetic resonance imaging (MRI) and computed tomography (CT) findings, followed by histopathologic demonstration of noncaseating granulomatous inflammation without evidence of malignancy or infection, illustrates the diagnostic complexity of vertebral sarcoidosis and the importance of tissue confirmation when imaging findings are inconclusive.

## Case presentation

A 51-year-old female patient with a past medical history significant for immune thrombocytopenic purpura, hypertension, chronic kidney disease, and prior resected melanoma presented following incidental findings on routine screening mammography. Family history was notable for testicular cancer in a brother and breast cancer in her paternal grandmother. She denied tobacco use.

Screening mammography revealed axillary lymphadenopathy, which was further evaluated with ultrasound-guided biopsy. Histopathological analysis demonstrated noncaseating granulomas without evidence of mycobacterial or fungal infection.

Subsequent chest radiography revealed bilateral hilar lymphadenopathy (Figure 1). At that time, the patient denied respiratory symptoms, including dyspnea, cough, wheezing, or chest discomfort. Pulmonary function testing demonstrated a moderate obstructive defect with normal lung volumes and mildly elevated diffusing capacity. CT of the chest showed bilateral nodular opacities, including nodules greater than 1 cm in the left lower lobe and right upper lobe. 

**Figure 1:**
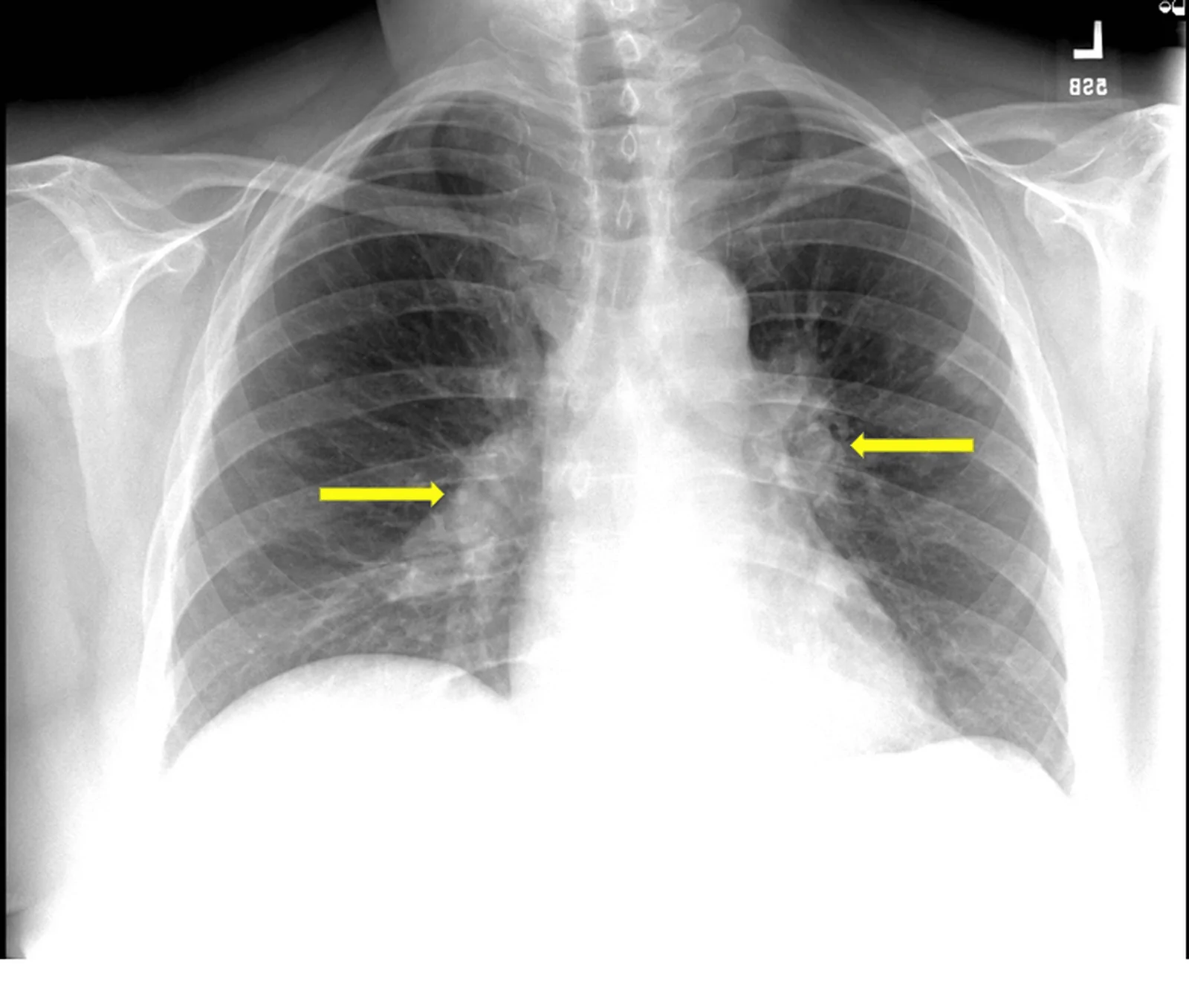
Chest radiograph demonstrating bilateral hilar prominence Chest radiograph demonstrating bilateral hilar prominence (yellow arrows), consistent with pulmonary involvement of sarcoidosis

The patient subsequently underwent bronchoscopy with endobronchial ultrasound (EBUS) and biopsy, which revealed respiratory epithelial cells with epithelioid histiocytes and multinucleated giant cells consistent with granulomatous inflammation (Figure 2). No evidence of malignancy was identified. She was treated with a course of prednisone followed by taper.

**Figure 2:**
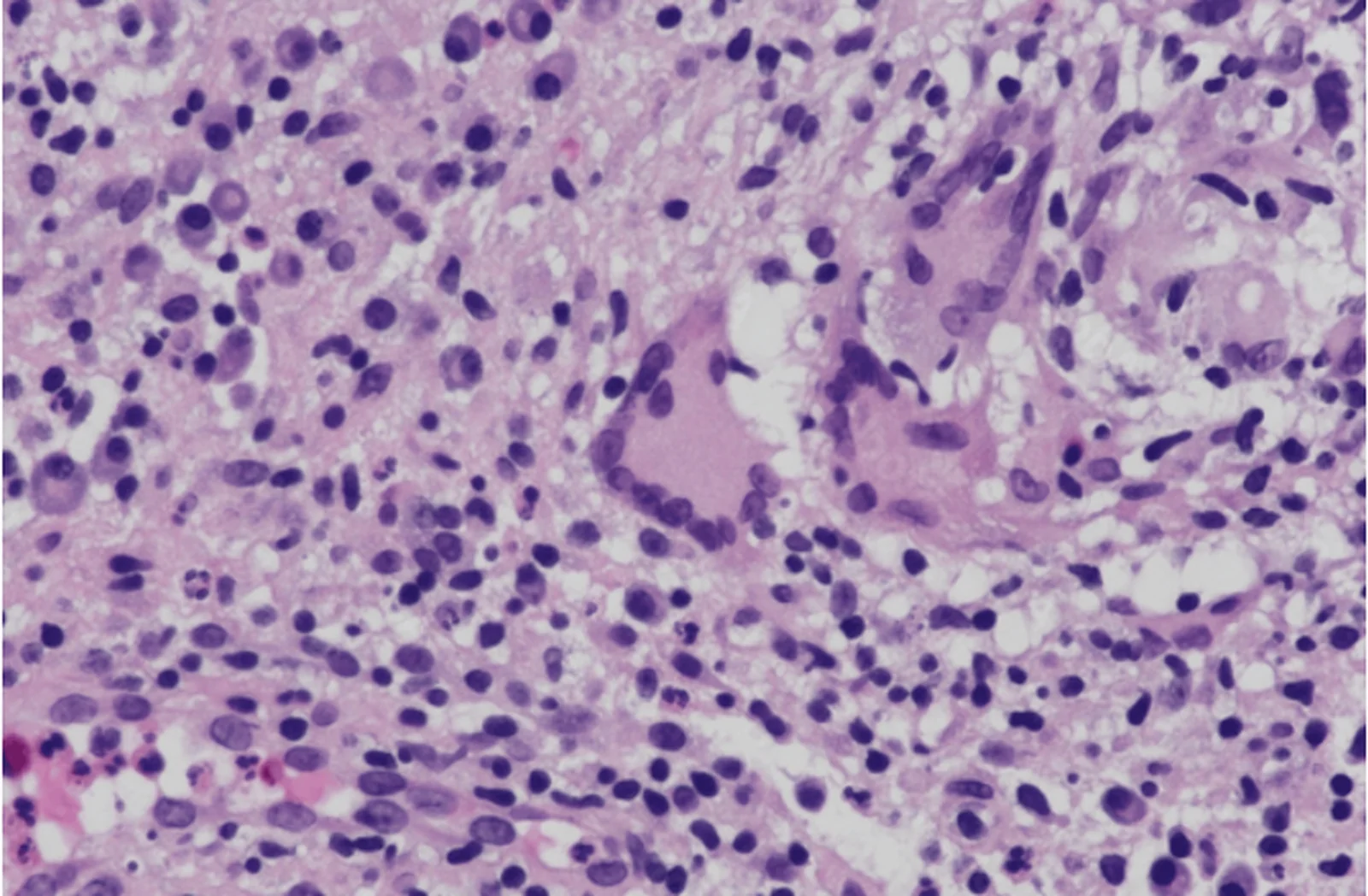
Histopathologic examination demonstrating nonnecrotizing granulomatous inflammation Histopathologic examination of the lung biopsy showing nonnecrotizing granulomatous inflammation composed of epithelioid histiocytes and multinucleated giant cells (hematoxylin and eosin stain, ×40 magnification)

Later in the clinical course, the patient developed upper respiratory symptoms and was treated with multiple courses of antibiotics. Follow-up pulmonary function testing demonstrated a 24% decline in both forced vital capacity (FVC) and forced expiratory volume in one second (FEV1), with slightly reduced lung volumes and preserved diffusing capacity.

Approximately 15 months after the pulmonary diagnosis, the patient developed acute back pain following a fall at home. At the time of this presentation, she was not receiving corticosteroid therapy. The pain progressively worsened with movement, including walking, sitting, and standing. She denied prior back pain or constitutional symptoms such as fever, weight loss, or night sweats. Physical examination revealed tenderness over the lower thoracic spine with pain on range of motion.

Thoracic spine radiographs demonstrated a 14-degree rightward curvature in the mid-thoracic spine (T5-T8) and a 14-degree thoracolumbar curvature (T8-L1), with minimal loss of vertebral height at a mid-thoracic level, likely T9 (Figure 3). 

**Figure 3:**
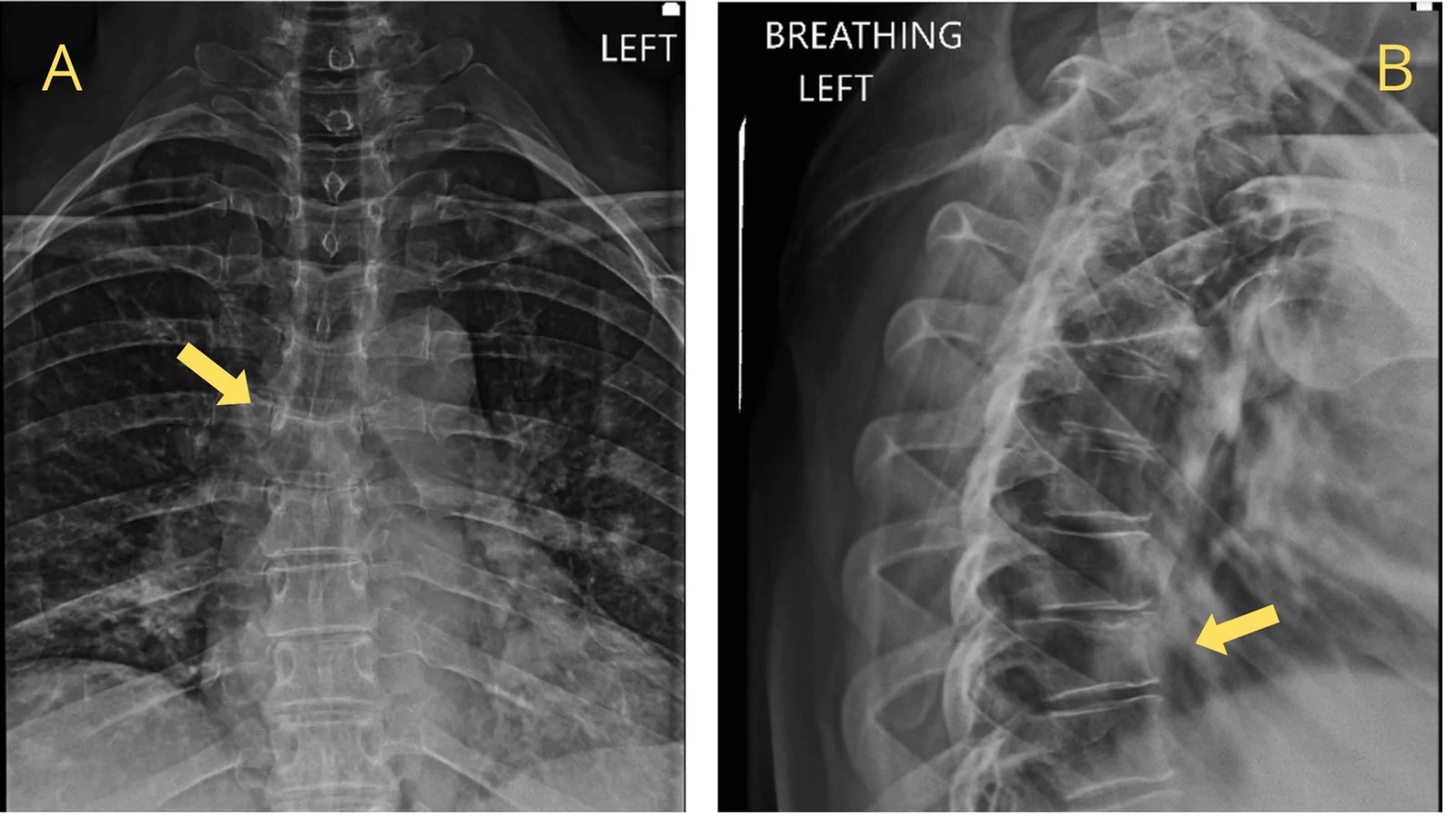
Thoracic spine radiographs demonstrating thoracic and thoracolumbar curvature T: thoracic vertebra; L: lumbar vertebra Anteroposterior (A) and lateral (B) thoracic spine radiographs demonstrating a 14-degree rightward mid-thoracic curvature (T5-T8) and a 14-degree thoracolumbar curvature (T8-L1), with minimal loss of vertebral height at a mid-thoracic level, likely T9. The arrows indicate the rightward mid-thoracic curvature (A) and minimal vertebral height loss (B)

MRI of the thoracic spine revealed superior endplate compression fractures at T7 and T9, more pronounced at T9, along with multifocal indeterminate marrow lesions. Notable lesions included a 1.5 cm lesion at L1, a 2 cm lesion at T11, and additional smaller lesions at T5 and T8. These findings raised concern for metastatic disease, particularly in the context of prior melanoma (Figure 4). 

**Figure 4:**
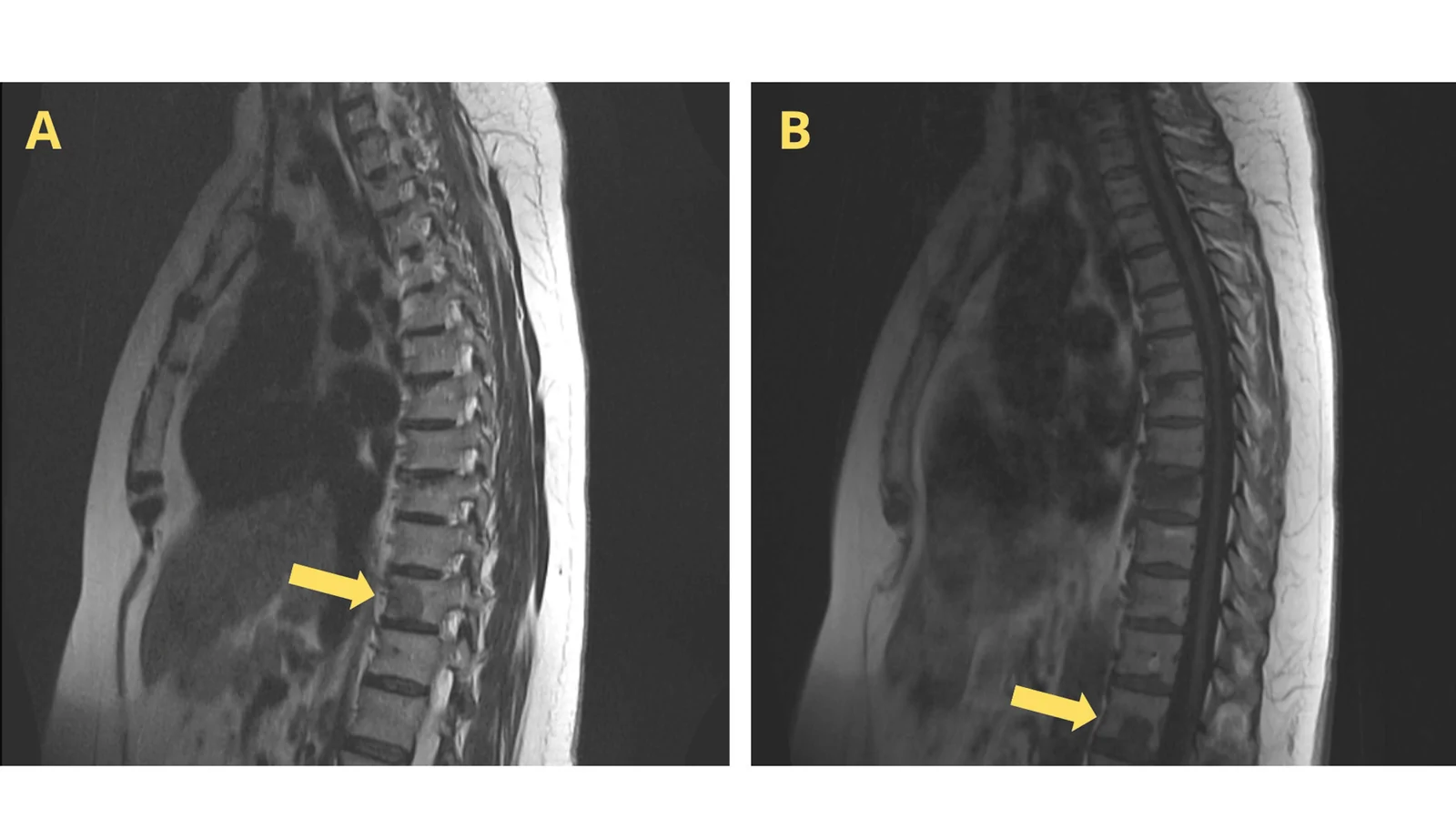
Thoracic spine MRI demonstrating multifocal vertebral marrow lesions MRI: magnetic resonance imaging (A) Sagittal magnetic resonance image demonstrating a focal area of low T1 signal intensity involving the T11 vertebral body (yellow arrow). (B) Sagittal magnetic resonance image demonstrating a focal area of low T1 signal intensity involving the L1 vertebral body (yellow arrow)

The patient was evaluated by oncology, and contrast-enhanced CT imaging of the thoracic and lumbar spine was obtained. CT imaging demonstrated incomplete healing of T7 and T9 compression fractures without features of pathological fracture and failed to identify corresponding abnormalities to the marrow lesions observed on MRI. No lesions were amenable to CT-guided biopsy (Figure 5). 

**Figure 5:**
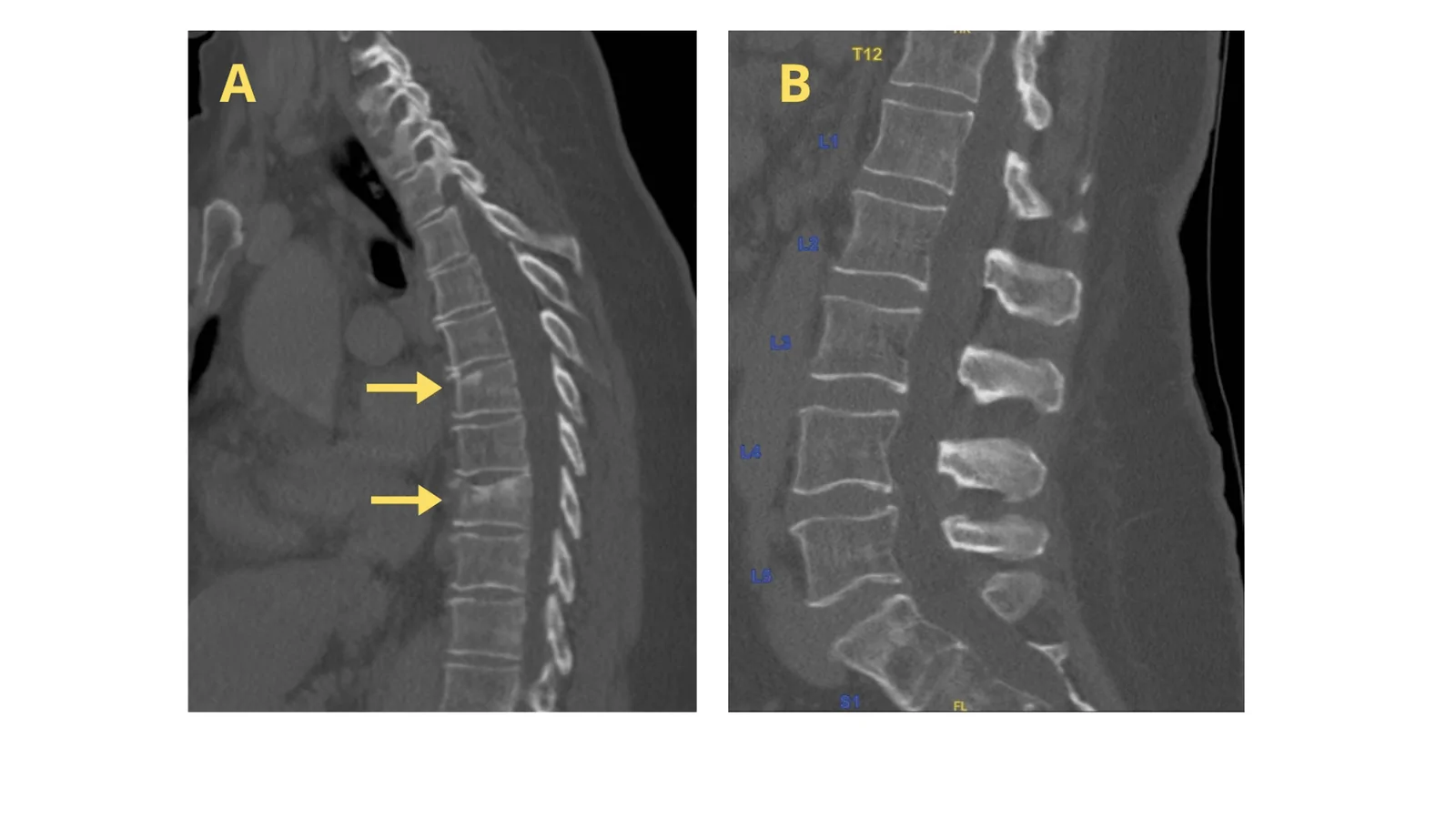
CT scan of the thoracic and lumbar spine showing lack of corresponding lesions CT: computed tomography; MRI: magnetic resonance imaging (A) Sagittal CT of the thoracic spine demonstrating compression fractures at T7 and T9 (yellow arrows), without corresponding structural abnormalities at the sites of the marrow lesions identified on MRI. (B) Sagittal CT of the lumbar spine demonstrating no corresponding structural abnormality at the L1 vertebral body, with vertebral levels labeled for anatomical localization

Given the patient’s history of melanoma and the multifocal vertebral lesions on MRI, metastatic disease was the primary concern. However, the differential diagnosis also included hematologic malignancy, infectious etiologies, and osseous sarcoidosis, particularly given the discordant CT findings.

Laboratory evaluation revealed mildly elevated serum calcium (10.7 mg/dL; reference range: 8.6-10.4 mg/dL), consistent with known metabolic manifestations of sarcoidosis. Serum protein electrophoresis demonstrated a mildly increased gamma fraction, and serum free light chain analysis showed elevated kappa and lambda chains.

Given persistent diagnostic uncertainty, a bone biopsy of the T11 vertebra was performed. Histopathological examination demonstrated benign bone with noncaseating granulomatous inflammation consistent with sarcoidosis. Acid-fast bacilli and fungal stains were negative, and no evidence of malignancy was identified (Figure 6).

**Figure 6:**
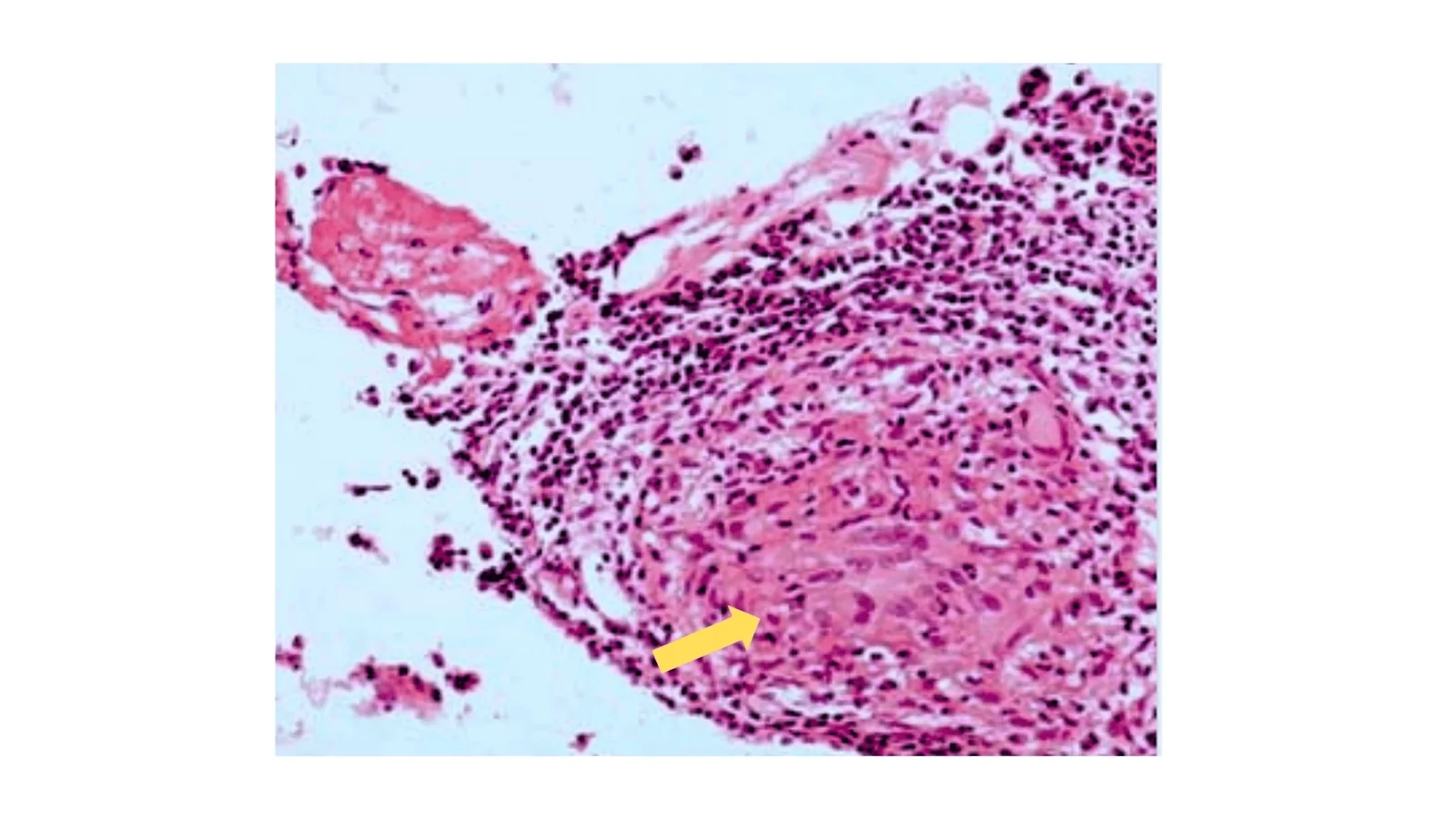
Histopathologic examination demonstrating nonnecrotizing granulomatous inflammation Histopathologic examination of the T11 vertebral body showing nonnecrotizing granulomatous inflammation consistent with sarcoidosis (yellow arrow). Hematoxylin and eosin stain, 10× magnification

The patient was subsequently evaluated by rheumatology and initiated on corticosteroid therapy followed by mycophenolate mofetil. After two months, she reported improved energy levels and decreased fatigue, with gradual improvement in back pain. Due to gastrointestinal side effects, mycophenolate mofetil was discontinued and replaced with methotrexate while continuing prednisone taper. Follow-up imaging demonstrated no new vertebral abnormalities, and previously noted compression fractures appeared chronic. The patient continued to demonstrate clinical improvement.

## Discussion

Sarcoidosis is a multisystem granulomatous disorder with heterogeneous clinical manifestations, most commonly involving the lungs and intrathoracic lymph nodes [1,2]. However, extrapulmonary disease may affect nearly any organ system and can present with a wide range of clinical manifestations, often resulting in diagnostic challenges.

Osseous involvement in sarcoidosis is uncommon, reported in approximately 3-13% of patients, and vertebral involvement is particularly rare [3]. When present, skeletal sarcoidosis may manifest with lytic, sclerotic, or mixed lesions, frequently indistinguishable from metastatic disease on advanced imaging modalities [5].

This case underscores several key diagnostic challenges. First, the patient’s prior history of melanoma significantly increased the pretest probability of metastatic disease, making the radiologic findings highly concerning. Second, MRI demonstrated multifocal vertebral marrow lesions suggestive of malignancy, whereas CT imaging failed to demonstrate corresponding structural abnormalities. MRI is particularly sensitive for detecting bone marrow abnormalities, whereas CT provides complementary assessment of cortical and structural bone changes. Consequently, vertebral sarcoidosis may demonstrate discordant findings across imaging modalities, further complicating its distinction from metastatic disease [4,6].

An additional supportive finding was mild hypercalcemia, a recognized metabolic complication of sarcoidosis resulting from dysregulated extrarenal production of 1,25-dihydroxyvitamin D by activated macrophages within granulomas [7].

Similar diagnostic challenges have been reported in the literature. Moore et al. evaluated MRI features of osseous sarcoidosis and metastatic bone disease and found that routine MRI could not reliably distinguish between the two entities, highlighting the limitations of imaging alone in this setting [8]. More recently, Bastidas et al. described osseous sarcoidosis presenting with multiple lytic and blastic lesions involving the axial skeleton that initially raised concern for metastatic disease [9]. These findings closely parallel the present case, in which multifocal vertebral marrow abnormalities in a patient with a history of melanoma strongly suggested metastatic recurrence until histopathological examination demonstrated noncaseating granulomatous inflammation.

The differential diagnosis in this case was broad and included metastatic melanoma, hematologic malignancy, infectious etiologies such as tuberculosis or fungal disease, and other granulomatous conditions. Given the high-risk clinical context, tissue diagnosis was essential. Vertebral biopsy ultimately confirmed noncaseating granulomatous inflammation without evidence of malignancy or infection, establishing the diagnosis of osseous sarcoidosis.

Importantly, this case highlights that sarcoidosis may initially present as an incidental finding, as seen in this patient whose diagnosis was prompted by abnormal screening mammography rather than respiratory symptoms. The subsequent development of vertebral lesions further illustrates the dynamic and multisystem nature of the disease.

From a management perspective, corticosteroids remain the cornerstone of therapy, with immunosuppressive agents such as methotrexate or mycophenolate mofetil used in patients with refractory disease or intolerance to steroids [7]. The patient’s clinical improvement following immunosuppressive therapy further supports the diagnosis and underscores the importance of early recognition and treatment.

Overall, this case emphasizes the necessity of maintaining a broad differential diagnosis when evaluating vertebral lesions, particularly in patients with a history of malignancy, and reinforces the critical role of histopathological confirmation in avoiding misdiagnosis and inappropriate management.

## Conclusions

In patients with a history of malignancy, new vertebral lesions should not be presumed to represent metastatic recurrence solely on the basis of imaging. This case demonstrates that vertebral sarcoidosis can closely mimic metastatic melanoma, particularly when MRI reveals multifocal marrow abnormalities without corresponding structural changes on CT. When imaging findings are discordant or atypical, tissue biopsy should be pursued before initiating oncologic treatment. The key clinical lesson is that histopathological confirmation can prevent misdiagnosis and unnecessary cancer-directed therapy in patients with vertebral lesions and a competing granulomatous disease.
